# The Picture Palace and the Spread of Disease

**Published:** 1914-04-25

**Authors:** 


### The Picture Palace and the Spread of Disease.

The mushroom-like growth of "picture palaces" and their immense popularity with the proletariat have had unforeseen results in promoting the spread of epidemic diseases. Dusty, crowded with children, and with sunlight rigidly excluded, some of these halls are veritable hot-beds of infection; and the closing of schools when an epidemic occurs makes matters worse instead of better, because the children all flock to the "pictures." A careful consideration by Dr. Alexander Graham of the epidemiology of mumps appears in the *British Journal of Children's Diseases*, and leads him to the following conclusions: An epidemic of mumps presents all the features of an epidemic of any other infectious fever. Malignant cases may occur, as in scarlatina, at the end of an epidemic. Members of the same family should attend the same school, and all children should continue to attend the schools in their own districts. The control of an epidemic by closing a school, or part of a school, is becoming increasingly difficult owing to the cinematograph shows. As soon as any suspicion of an infectious disease presents itself to a teacher, the school medical service should at once be put in operation to control an epidemic; no epidemic of a supposed mild disease should be looked upon lightly. Every possible means should be employed to avoid the absence of children from school; and the principal weapon in this direction is the school medical service.

On previous occasions we have drawn attention to this point, and also to the risks, from a public health point of view, inherent in the construction and design of the picture palace itself. The ventilation is frequently atrocious, and the design seems too often to have only the one object of crowding together the greatest possible number in the least possible space. It would, in fact, be a good thing if the care now taken by the licensing authorities over the number of exits and entrances, and in some degree over risks of fire, were extended to cover the equally grave risks of the present picture palace as a centre of infection.

